# Steps Forward for Cardio-Kidney-Metabolic Research in Transplant Recipients

**DOI:** 10.34067/KID.0000000796

**Published:** 2025-05-29

**Authors:** Kristin K. Clemens, Louise M. Moist

**Affiliations:** 1 Division of Endocrinology and Metabolism, Department of Medicine, Western University, London, Ontario, Canada; 2 Department of Epidemiology and Biostatistics, Western University, London, Ontario, Canada; 3 Lawson Research Institute, London, Ontario, Canada; 4 ICES, Ontario, Canada; 5 St. Joseph's Health Care London, London, Ontario, Canada; 6 Division of Nephrology, Department of Medicine, Western University, London, Ontario, Canada; 7 London Health Sciences Centre Research Institute, London, Ontario, Canada; 8 London Health Sciences Centre, London, Ontario, Canada

**Keywords:** diabetes mellitus, kidney transplantation, cohort studies

Glucagon-like peptide 1 receptor agonists (GLP-1RAs) have revolutionized the treatment of patients with metabolic disease. In large randomized controlled trials (RCTs) of participants with type 2 diabetes, nontransplant CKD, and obesity, GLP-1RAs have been proven to improve glycemic control; promote weight loss; reduce damage to the heart, brain, and kidneys; and lower the risk of death.^1^

In their article, Kahwaji *et al.* report on 185 kidney transplant recipients with type 2 diabetes who were prescribed a GLP-1RA (mostly liraglutide) within the Southern California Kaiser Permanente integrated health care organization.^2^ Patients were predominately non-Caucasian (76%) and of female sex (56%). Investigators examined the effect of GLP-1RA on hemoglobin A1c (HbA1c), body mass index (BMI), eGFR, and immunosuppressant levels every 3 months, until last transplant follow-up (median of 272 days).

There were promising short-term outcomes observed; the use of GLP1-RAs was associated with a reduction in HbA1c (8%–7.3%, *P* < 0.001) and BMI (35.2–33.2 kg/m^2^, *P* < 0.001) to the last follow-up. There was no change in eGFR or tacrolimus levels observed, and there was no graft failure to the end of follow-up. The authors suggest that GLP-1RAs should be considered for weight loss and diabetes control in kidney transplant recipients.

Kahwaji's findings are important and do align with the results of a recent systematic review (SR) and meta-analysis of nine observational cohort studies of GLP-1RA in kidney transplant recipients published in 2024.^3^ In the SR, Krisanapan *et al.* found that GLP-1RAs helped to reduce HbA1c (mean difference, −0.85%; 95% confidence interval, −1.4% to −0.28%), and BMI with stability in eGFR and creatinine observed over time. However, observational cohort studies, including the current report, are not without methodologic limitations. This study was small, single centered, had no comparator, and was subject to significant confounding (confounding by indication in particular). Only tacrolimus interactions were considered, excluding other immunosuppressants such as mycophenolate. Exploring the effect of GLP-1RAs on gastrointestinal side effects would have been important. In CKD and transplant populations, the risk of nausea and vomiting is high (17.6% of patients had gastrointestinal side effects in the aforementioned SR).^3,4^ Understanding the risk of GLP-1RA when coprescribed with insulin and other antihyperglycemic medications is also important given the risk of hypoglycemia with combination therapy.^5^ Besides eGFR effect, assessing the effect of GLP-1RAs on albuminuria would also be valuable, given the known kidney benefits of these drugs in nontransplant populations.^6^

Well-conducted multicentered RCTs of GLP-1RAs in kidney transplant recipients should continue to be encouraged. In Canada, the HALLMARK study will investigate the short-term efficacy of 12 weeks of dapagliflozin and semaglutide in 20 kidney transplant recipients with and without diabetes mellitus. Proposed outcomes include BP, heart, and kidney failure.^7^ The Semaglutide Treatment for Hyperglycaemia After Renal Transplantation study will examine the effect of oral semaglutide versus placebo on plasma glucose in 100 participants with hyperglycemia after kidney transplant.^8^

Future studies should also focus on longer term cardio (cardiovascular disease), kidney (graft outcomes), and metabolic outcomes (diabetes, weight) with use of GLP-1RAs, along with the safety of GLP-1RAs in transplant recipients (hypoglycemia, gastrointestinal side effects, and AKI).^9^ Special attention to the risk of infection and malignancy is essential given the use of immunosuppressant medications in this patient population.^10^ Future trials might also explore the optimal timing of GLP-1RA initiation, and how these medicines might be positioned alongside other therapies including sodium-glucose cotransporter 2 inhibitors in kidney transplant recipients. Clinical research should continue to prioritize inclusivity, ensuring diverse participation (*e.g*., women and non-Caucasian individuals), to enhance real-world applicability.

In summary, Kahwaji *et al.* share more evidence for the use of GLP-1RAs in kidney transplant recipients. The conduct of larger, long-term, inclusive, and rigorous RCTs needs to be encouraged.
